# A multicenter prospective study on the diagnostic performance of a new liquid rapid urease test for the diagnosis of *Helicobacter pylori* infection

**DOI:** 10.1186/s13099-017-0226-5

**Published:** 2017-12-22

**Authors:** Werner Dolak, Ceren Bilgilier, Alexander Stadlmann, Judith Leiner, Andreas Püspök, Wolfgang Plieschnegger, Franz Siebert, Friedrich Wewalka, Rainer Schöfl, Ursula Huber-Schönauer, Christian Datz, Susanne Biowski-Frotz, Christoph Högenauer, Christiane Schrutka-Kölbl, Athanasios Makristathis, Maximilian Schöniger-Hekele, Christoph Steininger, for the Austrian Helicobacter Pylori Study Group

**Affiliations:** 1 Gastroenterology and Hepatology, Internal Medicine III., Medical University of Vienna, Vienna, Austria; 2 Department of Medicine I, Infectious Diseases, Internal Medicine I, Medical University of Vienna, Währinger Gürtel 18-20, 1090 Vienna, Austria; 3 Internal Medicine, Ladislaus Batthyány-Strattmann Hospital Kittsee, Kittsee, Austria; 4 Internal Medicine II, Hospital of the Brothers of Saint John of God Eisenstadt, Eisenstadt, Austria; 5 Internal Medicine, Hospital of the Brothers of Saint John of God St Veit/Glan, St Veit, Austria; 6 Internal Medicine 4, Elisabethinen Hospital Linz, Linz, Austria; 7 Internal Medicine, Hospital Oberndorf, Oberndorf, Austria; 8 Ordination Dr. Susanne Biowski-Frotz, Vienna, Austria; 9 Gastroenterology and Hepatology, Internal Medicine, Medical University of Graz, Graz, Austria; 10 Endoskopie Schrutka, Vienna, Austria; 11 Clinical Microbiology, Medical University of Vienna, Vienna, Austria

**Keywords:** *Helicobacter pylori*, Rapid urease test, Endoscopy

## Abstract

**Background:**

*Helicobacter pylori* (*H. pylori*) causes a diversity of gastric diseases. Rapid urease tests (RUT) are well established for the point-of-care, invasive diagnosis of *H. pylori* infection. The study aimed to evaluate the diagnostic performance of a new liquid RUT, the preOx-HUT, within a prospective cohort of treatment-naïve patients.

**Methods:**

The multicenter prospective clinical trial was conducted at nine Austrian centers for gastrointestinal endoscopy. Patients referred for a diagnostic upper gastrointestinal endoscopy underwent gastric biopsy sampling for routine histological evaluation, and in parallel, the preOx-HUT. Histology served as reference standard to evaluate the diagnostic performance of the preOx-HUT.

**Results:**

From January 2015 to January 2016, a total of 183 consecutive patients (54 males and 129 females, median age 50 years) were included. Endoscopy revealed pathological findings in 149/183 cases (81%), which were mostly gastritis (59%) and gastro-esophageal reflux disease (27%). *H. pylori* infection was detected by histology in 41/183 (22%) cases. In relation to histology, the preOx-HUT had a sensitivity of 85%, a specificity of 94%, a positive predictive value of 80% and a negative predictive value of 96%. Performance of preOx-HUT was not affected significantly by concomitant PPI-use as present in 15% of cases (P = 0.73).

**Conclusions:**

This was the first study evaluating the preOx-HUT in a prospective, multicenter clinical setting. We found a high diagnostic accuracy for the point-of-care, invasive diagnostic test of *H. pylori* infection. Hence, this test may be a valuable diagnostic adjunct to the clinical presentation of patients with suspected *H. pylori* infection.

*Trial registration number* EK 1548/2014, Name of registry: Register der Ethikkommission der Medizinischen Universität Wien, URL of registry: https://ekmeduniwien.at/core/catalog/2012/, Date of registration: 24.09.2014, Date of enrolment of the first participant to the trial: 15.01.2015

**Electronic supplementary material:**

The online version of this article (10.1186/s13099-017-0226-5) contains supplementary material, which is available to authorized users.

## Background

*Helicobacter pylori* (*H. pylori*) is a Gram-negative, spiral-shaped bacterium that colonizes the stomach of up to 50% of the population worldwide [1]. *H. pylori* infection is associated with a variety of gastric diseases such as peptic ulcer disease, gastritis, mucosa-associated lymphatic tissue (MALT) lymphoma and gastric adenocarcinoma. *H. pylori* is one of three top causes of infection-associated cancer and the most relevant bacterial carcinogen in the gastrointestinal tract [2]. The indications for testing patients for *H. pylori* infection and antimicrobial treatment for eradication of *H. pylori* are clearly defined in the international Maastricht consensus guidelines and include primarily dyspeptic symptoms [3]. Evaluation for *H. pylori* infection is also important to treat infection-associated disorders such as gastric or duodenal ulcer disease, MALT-lymphoma, or persistent dyspepsia.

The diagnosis of *H. pylori* infection may be established with use of a variety of invasive and non-invasive tests (reviewed in [4]). Upper gastrointestinal endoscopy with histological evaluation of biopsies taken from the gastric body and antrum is the current reference standard for the detection of *H. pylori* infection. Specific stains such as Giemsa stain enhance the visibility of *H. pylori* on histological sections and consequently the sensitivity of the assay. The specificity of histology is somewhat limited by the occasional presence of non-pylori *Helicobacter* like organisms (HLO) such as *H. heilmannii*, which are morphologically indistinguishable from *H. pylori*. A higher specificity of *H. pylori* detection in biopsy samples may be attained with use of more elaborative and expensive tests such as immunohistochemistry, PCR, or bacterial culture. Culture of gastric biopsies for *H. pylori* has the added benefit of characterizing the antimicrobial susceptibility of clinical *H. pylori* strains—a procedure that is recommended after two treatment failures to tailor eradication regimens to the individual antimicrobial resistance pattern—but has the disadvantage of a low sensitivity [5].

Non-invasive tests for the diagnosis of *H. pylori* infection include ^13^C-Urea breath tests, *H. pylori*-specific serology, and stool antigen tests, which differ in diagnostic accuracy and clinical application. Breath and stool tests are useful to assess the success of antimicrobial eradication treatment, especially when invasive procedures would not be required or indicated (for example after resolution of symptoms in non-ulcer dyspepsia). Serology allows the evaluation for infection with *H. pylori* but may not allow differentiation between past and recent, active infection since *H. pylori*-specific IgG remain positive even after successful eradication of the pathogen [6].

In addition to these laboratory-based diagnostic assays, *H. pylori* infection may be also diagnosed with use of a point-of-care or bedside test—the rapid urease test (RUT). *H. pylori* genes code for bacterial urease, which is essential for metabolism of *H. pylori* and colonization of the gastric mucosa. The presence of this enzyme in a clinical sample may be visualized by hydrolyzing urea in a test medium to ammonia and carbon dioxide. The subsequent increase in pH is indicated by a color-based pH-indicator [7]. Multiple commercial RUT kits are available in different formats. The major advantage of this type of tests is the availability of a bed-side test result within minutes to a few hours which allows the clinicians to perform the test during or shortly after endoscopy. Moreover, the reported diagnostic accuracy is excellent with sensitivity rates of 80–90% and specificity rates well above 90% [8–10].

The aim of the present study was to evaluate the diagnostic accuracy of the preOx-Helicobacter urease test (HUT), a new liquid rapid urease test within a large, multicenter, prospective cohort of treatment-naïve patients. For this purpose, gastric biopsy samples from *H. pylori* therapy-naïve patients without previous gastroscopy in their history were tested in parallel by histology and preOx-HUT. We found that test performance of the preOx-HUT in therapy-naïve patients was comparable to that of similar tests evaluated in previous studies.

## Methods

This prospective, multi-center, observational clinical trial was conducted at nine geographically distant centers for gastrointestinal endoscopy in Austria, including two primary care institutions, five referral hospitals and two university-based centers. The study protocol was approved by the internal review board of the Medical University of Vienna (EK 1548/2014) and the Austrian Federal Office for Safety in Health Care. All adult patients referred for a diagnostic upper GI endoscopy were screened to participate in this study. A previous upper GI endoscopy as well as any prior eradication therapy for *H. pylori* were considered as exclusion criteria. Concomittant proton pump inhibitor (PPI) therapy was paused for a minimum of 2 weeks before endoscopy. Patients were included into the study after oral and written informed consent had been obtained. Study subjects underwent upper GI endoscopy with standard biopsy sampling of both antrum and body of the stomach (two biopsies from each site), processed for histological evaluation. Additionally, another biopsy was taken from each of these two locations and used for on-site rapid urease testing with the preOx-HUT (PreOx.RS GmbH, Limburg, Germany).

### Performing the preOx-HUT

All procedures of the preOx-HUT were done according to the manufacturer’s recommendation. In brief, gastric biopsies were transferred into the urease medium and incubated at room temperature for 10 min. The presence of *H. pylori* is indicated by a color change of the medium from yellow into violet. Test results were documented and compared with the histological evaluation to calculate the parameters of diagnostic accuracy for the preOx-HUT.

### Histological evaluation

Biopsy samples dedicated for histology were formalin fixed and paraffin embedded. Histo-morphologic assessment and evaluation for presence or absence of HLO was based on hematoxylin and eosin (H&E) stained sections and additionally, modified Giemsa staining was performed to identify colonization by *H. pylori* of the gastric mucosa. The pathologists evaluating the histological sections were blinded to the results of the preOx-HUT.

### Statistical assessment

Demographic data (age, sex), indication for upper gastrointestinal endoscopy, concomitant PPI treatment and the endoscopic results were reported descriptively. Defining the histological result as the reference standard, sensitivity, specificity, positive predictive value and negative predictive value were calculated for the preOx-HUT. Subgroups for concomitant PPI-use (yes/no) were analyzed regarding the diagnostic performance of the preOx-HUT using logistic regression analysis. A P value < 0.05 was considered significant. Based on a previously reported prevalence of *H. pylori* in Austria of 20% [11], we assumed a sample size of at least 150 subjects that have to be included into the study to ensure a sufficient amount of *H. pylori* positive cases for reliable calculation of diagnostic accuracy of the test. In view of the average patient frequency at the participating institutions we expected to recruit the required amount of study subjects within 1 year. All data analyses were performed using SPSS (version 23.0). All co-authors had access to the study data and reviewed and approved the final manuscript.

## Results

From January 2015 to January 2016, a total of 183 consecutive patients (54 males and 129 females, median age 50 years, range 18–92 years) were included in this study. Indications for gastroscopy were upper abdominal pain (38%) and reflux symptoms (25%), followed by cancer screening (15%) and dysphagia (5%). Rare causes included suspected celiac disease (3%), irritable bowel syndrome (3%), gastrointestinal bleeding (3%). Only a minority of patients had a concomitant PPI-therapy (28 cases, 15%) at the time of endoscopy. Endoscopy revealed pathological findings in 149/183 cases (81%), which were mostly gastritis (59%) and gastro-esophageal reflux disease (27%). An ulcer was found in 4%, a tumor in 2% of cases (Table 1, Additional file 1: Table S1).

**Table 1:** Study population

	*N*	%
Gender M:F	54:129	30:70
Median age	50 years (range 18–92 years)	N/A
Concomitant PPI therapy	28	15
Pathology at endoscopy	149	81
Ulcer disease at endoscopy	7	4
Tumor at endoscopy	4	2

Evaluation of the biopsies from antrum and corpus with use of histology showed the presence of HLO in 41/183 (22%) cases. Testing of biopsy samples with use of the preOx-HUT was positive in 44/183 (24%) of cases. Test results were congruent in 168/183 samples, 9/142 (6%) of the histology-negative samples gave a positive test result in the preOx-HUT and 6/41 (15%) of the histology-positive samples gave a negative test result in the preOx-HUT. The sensitivity of the preOX-HUT was accordingly 85%, the specificity was 94%, the positive predictive value 80%, and the negative predictive value was 96% (Table 2).

**Table 2:** Test results for *Helicobacter pylori*

	Histology		Total
	Negative	Positive	
preOx-HUT			
Negative	133	6	139
Positive	9	35	44
Total	142	41	183

Concomitant PPI-use did not show a significant correlation to the diagnostic accuracy of the preOx-HUT (P = 0.73).

## Discussion

This study reports on the largest clinical evaluation of the preOx-HUT in the context of a multi-center trial. The frequency of HLO as detected by histology was 22% which is comparable with previous data on the frequency of *H. pylori* in Austria. The preOx-HUT had an excellent specificity and a very good sensitivity in this study.

Factors affecting the diagnostic accuracy of the rapid urease test have been previously studied. Among others, a low density of the pathogen in the gastric mucosa can cause false negative results. This can be due to incomplete eradication therapy, for example, which is not applicable in the present study as patients with previous antibiotic treatment for *H. pylori* were not allowed to participate [12, 13]. Furthermore, a progression of the inflammation induced by *H. pylori* by means of an increase of gastric atrophy can lead to a decrease of detectable bacteria, especially in the antrum [14]. To overcome this limitation, biopsies were taken from different gastric sites including both gastric antrum and body which also reflects the reference standard of biopsy sampling for histological evaluation. Recently, this biopsy strategy was supported by a prospective study that reported a combined antral and corpus RUT to be superior to a single antral RUT [15]. Another factor that can influence the diagnostic accuracy of the RUT is hypochlorhydria, typically seen in PPI users. This condition can lead to false positive results since other bacteria with urease activity might be able to colonize the stomach [12]. In this sample, only a minority of patients had a concomitant PPI therapy which was paused 2 weeks prior to endoscopy. According to subgroup analysis it did not affect the diagnostic accuracy of the preOx-HUT. Finally, bleeding was reported to cause false negative results [16], but none of the patients in this study did present with signs of active gastrointestinal bleeding at the time of upper gastrointestinal endoscopy.

Sensitivity and specificity of the preOx-HUT observed in the present study are in line with previous publications on different RUT kits [17–19]. While the various test kits in the market share the principle of a chemical reaction based on the activity of the enzyme urease in the biopsy specimen, the configuration of kits is slightly different. In our opinion, the liquid design of the preOx-HUT is superior to so called dry RUTs as it is supporting our standard workflow of biopsy processing. The test kit comes with a rack to host eight different test tubes within the endoscopy room. This provides a safe and easy application of the specimen and is sufficient for the desired observation period of the test. In opposite to many other trials, the preOx-HUT was not observed any longer if it was still negative after a total time span of 3 h. This workflow, which is in line with the manufacturer’s instruction can be seen as a limitation of the study design since the sensitivity could have even improved at a later time point. However, from a clinical perspective, we decided not to extend the observation time since we felt that it would have been contradictive for the evaluation of a so called “rapid” urease test [19]. Defining histology as the only reference standard for the diagnosis of *H. pylori* can be seen as another shortcoming of the present study. It is known from previous data that even histology has not a perfect diagnostic accuracy for the detection of *H. pylori* [20]. Different *Helicobacter species*, such as *H. heilmannii* can cause false positive results at microscopy, which has to be kept in mind during interpretation of the present data. On the other hand, a recent study validating histology with a highly sensitive PCR assay for the detection of *H. pylori* found false negative histological results to be < 1% and histology even more sensitive than PCR [21].

In general, sensitivity rates of nearly 100% reported in some studies have to be interpreted with caution, especially in view of the bed-side design of a RUT. Patient selection (e.g. restriction of concomitant therapy or indication for gastroscopy) might be a key factor to explain such diagnostic performance [8, 9]. Contrary, the present, well-defined patient population, restricting eligible subjects only to those never treated against *H. pylori* and not having any previous upper gastrointestinal endoscopy, is a big advantage of this trial. Furthermore, involving study centers from different geographical regions in Austria as well as centers with different levels of specialization, from primary care up to university based centers, is another quality parameter, confirmed by an actual prevalence of *H. pylori* which is in line with previous data on the frequency of the pathogen in Austria [11].

Compared to other rapid tests in medicine, such as near-patient assays for the diagnosis of influenza virus infection, for example, where sensitivity ranges between 10 and 40%, depending on the cohort evaluated, the diagnostic accuracy of the different RUTs is very impressive [22]. Nevertheless, in view of the present data and according to previous recommendations, treatment decisions should always consider the pretest likelihood of *H. pylori* positivity according to the clinical appearance and the findings of white light endoscopy (e.g. duodenal ulcer disease). In case of an unclear clinical picture and a negative RUT, treatment decisions should also consider the results of other test modalities for *H. pylori*, such as histology [7]. Following this concept, eradication therapy will be initiated after a positive RUT in patients with typical symptoms and endoscopic proof of an ulcer disease, while it might be withhold until the histological result if endoscopy is normal and the RUT is negative.

## Conclusions

In conclusion, this prospective multi-center study performed in a representative, therapy-naïve Austrian patient population found a high diagnostic accuracy of the preOx-HUT to diagnose *H. pylori* on site. Nevertheless, the results of the preOx-HUT have always to be interpreted in correlation to the clinical and endoscopic picture before initiating antibiotic treatment.

## Additional file

**Additional file 1.** **Table S1.**
