# Supplementary material for: A multicenter prospective study on the diagnostic performance of a new liquid rapid urease test for the diagnosis of Helicobacter pylori infection
Source: Gut Pathog. 2017 Dec 22;9:78. doi: 10.1186/s13099-017-0226-5 (PMC5740919; doi:10.1186/s13099-017-0226-5)
Supplement: Supplementary file 1 — Additional file 1. Table S1. [file 13099_2017_226_MOESM1_ESM.pdf]

Pat. ID	Region	Sex	Age	Indication	Underlying Disease	Concomittant Medication	Endoscopy result	Other result	HUT	Histol.
1	1	2	89	1	1,2	1	3		0	0
2	1	2	25	2	0	2	0		0	0
3	2	1	54	7	0	0	5	Portal hypertension	1	0
4	2	1	53	7	0	0	5	Hiatal hernia	1	1
5	2	2	23	6	0	0	1		0	0
6	2	1	55	7	0	0	1		1	1
7	2	1	50	7	0	0	1		0	0
8	2	1	61	7	0	0	0		1	0
9	2	1	47	7	0	0	1, 2		0	0
10	2	1	67	7	1	0	1, 5	Angiodysplasia	1	1
11	2	1	51	0	0	0	1, 2, 5	Hiatal hernia	0	0
12	2	1	55	7	1	1	1, 2, 3, 5	Hiatal hernia	0	1
13	2	1	51	7	0	0	1, 2, 5	Hiatal hernia	0	0
14	2	1	50	1	0	0	1, 5	Hiatal hernia	0	0
15	2	1	58	9	1	0	1		0	0
16	2	2	62	3	0	0	1		0	0
17	2	2	73	7	0	1	0		1	1
18	2	2	64	1	0	0	1		1	1
19	2	1	33	3	0	0	0		1	1
20	2	1	50	1	0	0	2		0	0
21	2	2	76	2	0	0	1, 2		0	0
22	2	2	73	1	0	2	0		0	1
23	2	2	19	1	0	0	1		0	0
24	2	2	59	7	0	1	0		0	0
25	2	1	51	2	0	0	0		0	0
26	2	2	74	3	0	0	5	Hiatal hernia	1	1
27	2	2	66	3	0	0	5	Hyperplastic polyps	0	0
28	2	2	63	2	0	3	1		0	0
29	2	1	28	1	0	0	0		0	0
30	2	2	66	1	0	0	1, 2, 5	Hiatal hernia	0	1
31	2	2	50	3	0	2	0		0	0

32	2	2	52	3	0	0	5	Hiatal hernia	0	0
33	2	2	40	1	0	2	0		0	0
34	2	1	56	3	2, 5	0	1, 2, 5	Schatzki's ring	0	0
35	2	2	27	3	0	0	5	Hiatal hernia	0	0
36	2	2	44	8	0	0	0		1	0
37	2	2	80	8	6	2	5	Hiatal hernia	0	0
38	2	1	58	1	0	0	1		0	0
39	2	2	34	3	0	0	5	Hyperplastic polyps	0	0
40	2	2	49	3	0	0	0		1	1
41	2	2	27	1	0	0	0		1	0
42	2	2	23	9	0	3	0		1	0
43	2	1	71	0	2	2, 3	2		0	0
44	2	2	52	10	0	0	0		1	1
45	2	2	33	1	0	0	0		0	0
46	2	2	36	5	0	2	1		1	1
47	2	2	59	3	0	0	1, 5	Hiatal hernia	0	0
48	2	2	57	7	0	0	5	Hiatal hernia	0	0
49	2	2	41	7	0	0	1, 5	Hiatal hernia	1	1
50	2	2	30	1	0	2	0		0	0
51	2	2	25	8	0	0	1		1	1
52	2	2	70	2	0	0	1, 5	Hiatal hernia	1	1
53	2	2	75	3	0	0	0		0	0
54	2	2	49	1	6	0	1		0	0
55	2	2	75	0	0	0	2, 5	Hiatal hernia	0	0
56	2	2	30	1	0	0	5	Hiatal hernia	0	0
57	2	2	52	1	0	2	1		1	0
58	2	2	58	1	1	0	1, 5	Hiatal hernia	0	0
59	2	2	67	3	0	2	2		0	0
60	2	1	53	3	0	0	1, 5	Hiatal hernia	0	1
61	2	2	34	5	0	0	5	Villous atrophy	0	1
62	2	2	35	4	6	1	5	Hiatal hernia	0	0
63	2	2	76	10	0	0	5	Hiatal hernia	0	0

64	2	2	57	1	0	0	0		1	1
65	2	1	40	0	0	0	1		0	0
66	2	2	42	10	0	0	1		0	0
67	2	2	40	3	0	4	1, 5	Hiatal hernia	0	0
68	2	1	30	10	0	1	1		1	1
69	2	1	42	1	0	0	1		0	0
70	2	2	63	3	0	0	4		1	1
71	2	2	38	1	0	0	0		0	0
72	2	2	63	1	6	1	1		0	0
73	2	2	49	10	0	0	5	Hiatal hernia	0	0
74	2	1	54	3	0	0	1, 2, 5	Hiatal hernia	0	0
75	2	2	49	1	0	0	1		1	1
76	2	2	34	4	0	0	0		0	0
77	2	2	59	7	0	0	0		0	0
78	2	2	33	1	0	0	0		0	0
79	2	2	49	1	0	2	1		0	0
80	2	2	44	3	0	0	0		0	0
81	2	2	24	1	0	0	5	Villous atrophy	0	0
82	1	2	76	7	1, 3	2	1		0	0
83	1	2	38	3	0	2	1, 2		0	0
84	1	2	31	1	0	0	0		0	0
85	1	2	30	3	0	0	1		0	0
86	1	2	70	3	0	0	1, 2		1	0
87	1	2	25	1	0	0	1, 2		0	0
88	1	1	25	1	0	0	1		0	0
89	1	1	73	3	0	0	1, 2		0	0
90	1	2	42	1	0	0	1		0	0
91	1	2	59	3	0	0	1		0	0
92	1	2	59	3	0	0	1, 2		0	0
93	1	2	22	1	0	0	1		0	0
94	1	1	31	1	0	0	1		0	0
95	1	2	29	1	0	2	1		0	0

96	1	2	38	1	0	0	0		0	0
97	1	1	67	2	0	0	1, 5	Esophageal divertic.	1	1
98	1	2	22	1	0	2	1, 2		0	0
99	1	2	42	8	0	0	0		1	1
100	1	2	33	3	0	0	1, 3		0	0
101	1	2	28	1	6	0	5	Plaque esophagus	0	0
102	1	2	53	1	0	0	1, 3		1	1
103	1	1	22	1	0	0	2		0	0
104	1	2	49	3	0	2	1		0	0
105	1	2	26	1	0	0	1		0	0
106	1	2	65	3	1	2	1, 2, 3, 5	Portal hypertension	1	1
107	1	2	61	3	0	0	1		0	0
108	1	2	59	5	2	3	1, 2		0	0
109	1	2	68	2	0	1	1, 2		1	0
110	1	2	20	1	0	0	1		0	0
111	1	2	36	1	0	0	2		0	0
112	1	2	41	1	0	0	0		0	0
113	1	1	18	1	0	0	1		0	0
114	1	2	18	3	0	2	1		0	0
115	1	2	38	1	0	0	1		0	0
116	1	1	35	1	0	0	1		1	1
117	1	1	24	1	0	1	2		0	0
118	1	1	54	3	0	0	1, 2		0	0
119	1	2	40	1	0	2	1		1	1
120	1	2	34	5	0	2, 3, 4	1		1	1
121	1	2	19	1	0	2	1		0	0
122	1	2	19	1	0	0	0		0	0
123	1	2	22	7	0	2	5	Hiatal hernia	0	0
124	1	2	54	3	0	0	1		0	0
125	1	2	60	3	0	0	1, 2		0	0
126	1	1	57	1	0	0	1, 2, 5	Hiatal hernia	1	1
127	1	2	57	3	0	0	2, 5	Hiatal hernia	0	0

128	1	2	30	1	0	0	1		1	0
129	1	2	57	3	0	2	1, 5	Hiatal hernia	0	0
130	1	1	30	1	0	0	1, 5	Hiatal hernia	0	0
131	1	2	54	1	0	1	1, 2		0	0
132	1	2	34	1	0	2	1		0	0
133	1	2	18	1	0	0	0		0	0
134	1	2	54	3	0	0	1, 2		1	1
135	1	2	27	1	3	0	1		0	0
136	3	1	55	1	0	0	1, 2, 5	Esophageal varices	0	0
137	3	2	37	0	0	0	0		0	0
138	3	2	48	6	0	0	2		0	0
139	3	2	78	2	1, 4	2	1		0	0
140	3	2	51	7	0	0	2, 4		0	0
141	3	2	36	1	0	0	1		0	0
142	3	2	73	1	0	0	5	goose pimple stom.	0	1
143	3	2	57	7	3	0	1, 5	Angiodysplasia	0	0
144	3	2	31	6	1, 6	3	5	Hyperplastic polyps	0	0
145	3	2	27	1	0	0	5	Hyperplastic polyps	0	0
146	3	2	18	1	0	2	1		0	0
147	3	1	51	7	0	0	1, 2		0	0
148	3	1	49	3	0	1	1		0	0
149	3	2	35	3	0	0	2		1	1
150	3	1	50	7	0	0	5	Hiatal hernia	0	0
151	3	1	46	1	0	0	2		0	0
152	3	1	74	3	0	0	1, 2		0	0
153	3	1	74	8	0	0	2		0	0
154	3	2	22	9	0	0	1		1	1
155	3	2	75	10	0	0	1, 5	Hiatal hernia	1	1
156	3	1	35	5	0	0	1, 2		0	0
157	3	2	18	1	3	0	5	Erosions	0	0
158	3	1	63	7	1	0	5	Hiatal hernia	0	0
159	3	1	56	7	0	3	1, 2, 5	Barrett's esophagus	0	0

160	3	1	40	3	0	0	2		0	0
161	3	2	53	1	0	0	1, 5	Angiodysplasia	0	0
162	3	2	24	9	0	0	0		0	0
163	3	2	92	2	0	0	1, 2		0	0
164	3	1	45	1	0	0	1, 2		0	0
165	3	2	66	10	0	0	1, 2		1	1
166	3	2	72	1	0	0	1, 5	Hiatal hernia	0	0
167	3	1	69	10	0	0	1, 5	St.p. Billroth II	0	0
168	3	2	65	3	0	0	1, 2		0	0
169	3	1	49	9	0	0	1		0	0
170	3	2	75	7	1	0	1, 5	Hiatal hernia	0	0
171	3	2	60	7	6	0	5	Hiatal hernia	0	0
172	3	1	78	3	0	0	1, 2		0	0
173	3	1	62	3	1	2	1		0	0
174	3	2	65	7	0	0	1, 2		0	0
175	3	1	42	3	0	0	1, 4, 5	Hiatal hernia	1	1
176	3	2	22	0	0	0	0		0	0
177	3	2	19	5	0	0	0		0	0
178	3	2	79	9	0	0	4, 5	Hiatal hernia	1	1
179	3	1	63	7	0	0	1, 2, 5	Hiatal hernia	0	0
180	3	1	47	1	0	0	1, 2, 5	Hiatal hernia	1	1
181	3	2	54	3	0	2	1, 3, 5	Zenker's diverticul.	1	1
182	3	2	36	1	0	0	1		0	0
183	1	2	47	1	0	0	1, 3		1	1

Legend

Region
1=East
2=West
3=South

Sex
1=male
2=female

Indication
0=Unknown
1=Abdominal pain
2=Dysphagia
3=Reflux
4=Anemia
5=Celiac disease?
6=Inflammatory Bowel Disease?
7=Screening
8=GI bleeding
9=Irritable Bowel Syndrome?
10=Various

Underlying disease
1=Diabetes
2=Hemato-oncological
3=Urogenital infections
4=Pneumonia
5=Chronic obstructive pulmonary disease
6=Inflammatory Bowel disease

Concomittant medication
1=Non steroidal anitinflammatory drugs
2=Proton pump inhibitor
3=Cortisone
4=Methotrexate

HUT / Histology
0=HP negative
1=HP positive

Endoscopy result
1=Gastritis
2=Esophagitis
3=Peptic ulcer
4=Tumor
5=Other
